# Occurrence of Deoxynivalenol and Deoxynivalenol-3-glucoside in Hard Red Spring Wheat Grown in the USA

**DOI:** 10.3390/toxins5122656

**Published:** 2013-12-18

**Authors:** Senay Simsek, Maribel Ovando-Martínez, Bahri Ozsisli, Kristin Whitney, Jae-Bom Ohm

**Affiliations:** 1 Department of Plant Sciences, North Dakota State University, PO Box 6050, Fargo, ND 58108, USA; E-Mails: maribel.ovando@ndsu.edu (M.O.-M.); kristin.whitney@ndsu.edu (K.W.); 2 Department of Food Engineering, College of Agriculture, Kahramanmaras Sutcu Imam University, Kahramanmaras 46060, Turkey; E-Mail: bozsisli@ksu.edu.tr; 3 USDA-ARS, Cereal Crops Research Unit, Harris Hall, Hard Red Spring and Durum Wheat Quality Laboratory, North Dakota State University, P.O. Box 6050, Fargo, ND 58108, USA; E-Mail: jae.ohm@ars.usda.gov

**Keywords:** deoxynivalenol, deoxynivalenol-3-glucoside, wheat, USA

## Abstract

Deoxynivalenol (DON) is a mycotoxin found in wheat that is infected with Fusarium fungus. DON may also be converted to a type of “masked mycotoxin”, named deoxynivalenol-3-glucoside (D3G), as a result of detoxification of the plant. In this study, DON and D3G were measured using gas chromatographic (GC) and liquid chromatography-mass spectrometry (LC-MS) in wheat samples collected during 2011 and 2012 in the USA. Results indicate that the growing region had a significant effect on the DON and D3G (*p* < 0.0001). There was a positive correlation between both methods (GC and LC-MS) used for determination of DON content. DON showed a significant and positive correlation with D3G during 2011. Overall, DON production had an effect on D3G content and kernel damage, and was dependent on environmental conditions during Fusarium infection.

## 1. Introduction

One of the most predominant and economically important mycotoxins affecting small-grain cereals, such as wheat, is deoxynivalenol (DON). DON is formed due to the presence of plant pathogenic fungi *Fusarium graminearum* and *F. culmorum*; which are responsible for the disease known as Fusarium head blight (FHB) [1]. The geographical distribution of these fungi is affected by climate. *F. culmorum* occurs more commonly in Europe while *F. graminearum* is common in Europe and North America [1,2]. The association between the FHB intensity level with DON accumulation in spring wheat using a meta-analysis has been reported in multiple studies [3]. The most important environmental factors causing FHB growth and biosynthesis of DON are water availability and temperature [4]. Minimum water activity values for growth and DON production appears to be limited at 0.93 under optimum temperature conditions (25 °C) [4,5]. Also, higher disease severity occurs if the wheat has been exposed to extended periods of wetness [1], which may result in higher DON production. On the other hand, *Fusarium* growth and DON production depends on the growth stages of the plant; in wheat higher damage occurs during flowering (anthesis) and shortly after flowering, the infection can also continue during grain maturation stage [1,6].

The presence of DON in wheat decreases grain quality by rendering the crop unsuitable and unsafe for food, feed and malting process. It is worthwhile noting that DON can have several side effects, such as feed refusal, vomiting, reduced weight gain, diarrhea, hemorrhage, skin lesions, growth depression and immunosuppression [7,8,9], which have a negative impact on human and animal health [6,10]. Also, DON affects the plant metabolism in wheat because it leads to the inhibition of germination and decreases plant growth. The plant starts to develop a detoxification mechanism in which DON is glycosylated into 3-β-d-glucopyranosil-4-deoxynivalenol (D3G) and stored inside the vacuole or cell wall to combat this situation [11,12]. This product is known as a “masked” mycotoxin, because one or more glucose molecules bind to the DON which reduces the toxicity in the plant and makes it unable to be detected by traditional methods for DON detection. D3G is less active as a protein biosynthesis inhibitor than DON [11]. There is a lack of information about the correlation between the DON and D3G production in wheat. Rasmussen *et al.* [13] found that the concentration of D3G is positively correlated with the increasing of DON content, which is similar to that observed by Lemmens *et al*. [14]. 

The toxicity of D3G in mammals is currently unknown. Berthiller *et al*. [15] reported that this “masked” mycotoxin resisted the *in vitro* acid conditions, indicating D3G cannot be hydrolyzed to DON in the stomach of mammals; nevertheless, it can be hydrolyzed during fermentation process by bacterial β-glucosidases in the colon. Later, it was demonstrated that D3G is partially absorbed in the gastrointestinal system of rats, but the majority of the D3G ingested was hydrolyzed in the digestion process and excreted in feces, which means that D3G is partially bioavailable in the gastrointestinal system of rats [16]. In Europe, the DON content of food, feed and unprocessed grains of undefined end-use collected in 21 European countries between 2007 and 2012 was recently published. It was found that the levels of DON in wheat, maize and oat may exceed the maximum limits for food or guidance values for feed. Due to the lack of data about D3G, this was not considered in the food safety assessment [17]. However, it may be necessary to analyze the DON and D3G content in wheat to have a clear idea about the total content of DON after the wheat is processed to different products. After determination of DON and D3G levels the assessment must be made to determine if the levels DON are within the permitted levels of DON approved by the Food and Drug Administration (FDA) and also to assess the safety of wheat-based products consumed in USA. The objective of this research was to analyze the DON and D3G content of hard red spring (HRS) wheat between 2011 and 2012 Crop Survey using gas chromatography (GC) and liquid chromatography-quadrupole time of flight mass spectrometry (LC-QTOF-MS) and find the correlation between the DON and D3G production. DON will be measured by both GC and LC-QTOF-MS to evaluate correlation between methods and determine the feasibility of the LC-QTOF-MS methodology for simultaneous measurement of DON and D3G.

## 2. Results and Discussions

### 2.1. Wheat Kernel Quality

**Figure 1:**
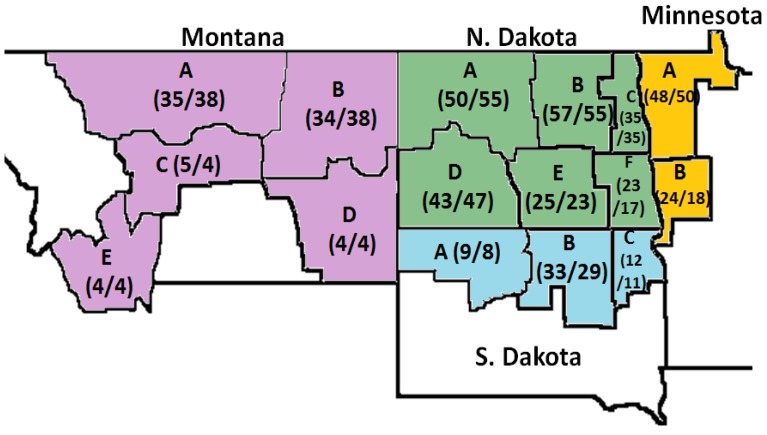
Distribution of hard red spring (HRS) wheat samples from the 2011 and 2012 Crop Surveys from Montana, North Dakota, Minnesota and South Dakota. **A**, **B**, **C**, **D**, **E** and **F**: regions in which the samples were collected from each state. The numbers inside the parenthesis represent the number of samples taken, from left to right: 2011 Crop Survey and 2012 Crop Survey.

The crop survey of HRS wheat was conducted in North Dakota (ND), South Dakota (SD), Montana (MT) and Minnesota (MN), which make up the primary HRS wheat growing region in the United States (US) (Figure 1). The Federal Grain Inspection Service (FGIS) use grading and non-grading factors to evaluate the conditions and quality of wheat. The HRS wheat kernel quality of the 2011 and 2012 crop surveys is presented in Table 1. The mean dockage percentage was the same in both survey crops. This factor does not affect the numerical grade but it is one important step in the grading process to eliminate all the material that is not wheat prior to determining the rest of grading and non-grading factors. Other than dockage, there were no differences observed in the grading factors percent shrunken and broken kernels, percent dark hard and vitreous (DHV) and test weight. However, the values of percent damage and percent total defects in the 2011 crop survey were higher than in the 2012 crop survey. According to the HRS wheat 2012 Regional Quality Report, there were differences in the environmental factors during planting, growing and harvest of crops of both years [18]. Higher temperatures and lower rainfall levels were observed in 2012, which decreased the disease pressure and percent damaged kernels, compared to 2011. These environmental factors in turn affected the wheat quality [19,20]. Among the non-grading factors (Table 1), the protein content is very important to determine the suitability of wheat in different final products. The protein percentage of both crop surveys was between the values established by the FGIS (13%–14%). HRS wheat is considered to have high and excellent protein quality for use in bread-baking. The samples from 2011 crop survey presented a lower falling number (386.3 s) compared to 2012 samples (429.3 s). Falling number is an indirect measurement of the α-amylase activity to detect sprout damage in wheat. This is accomplished by the measurement of changes in the physical properties of the starch portion of the wheat kernel caused by this α-amylase. A high enzyme activity indicates faster liquefaction and lower falling number. The falling number may be related to the higher percent damaged kernels and percent total defects presented in samples from 2011. Nevertheless, the falling number of both years were at least 350 s, which is the level at which grain is considered to be sound by the FGIS. Also, the 1000 kernel weight of 2011 crop survey samples was lower than 2012 crop survey samples. This means that the environmental factors (temperature, rainfall and moisture) could increase the percent damaged kernels and reduce the quality of wheat. Kernel quality was also affected by the production of mycotoxins, such as DON and its “masked” mycotoxin D3G, related to the occurrence of the FHB.

**Table 1:** Mean, standard deviation (SD), minimum (MIN) and maximum (MAX) values for wheat kernel quality characteristics for 2011 and 2012 crop survey samples.

Factors	2011				2012			
	Mean	SD	MIN	MAX	Mean	SD	MIN	MAX
Dockage (%) ^a^	1.1	1.4	0.0	13.4	1.1	1.2	0.0	13.3
Shrunken & broken ^a^	1.6	1.4	0.0	10.0	1.2	1.1	0.0	10.1
Damage (%) ^a^	0.5	0.8	0.0	10.6	0.1	0.2	0.0	2.8
Hard and vitreous kernel (%) ^a^	75.8	21.9	5.0	99.0	74.5	28.4	2.0	99.0
Test weight (lbs/bu) ^a^	60.0	2.4	52.5	65.7	60.9	1.9	53.9	65.1
Total defects (%) ^a^	2.1	1.7	0.1	13.1	1.3	1.1	0.0	10.1
Protein (12% mb) ^b^	14.8	1.3	10.2	18.6	14.6	1.4	10.3	20.1
Falling number (s) ^b^	386.3	44.1	226.0	621.0	429.3	48.8	238.0	654.0
1000 kernel weight (g) ^b^	26.7	4.1	16.8	40.0	29.1	4.1	16.6	44.1

Notes: ^a^ Grading factors used to evaluate the kernel quality to ensure general standards of acceptance in flour or semolina production; ^b^ Non-grade factors used to evaluate the kernel quality; mb = moisture basis.

### 2.2. Effect of the State and Region on DON, D3G and Damage Kernel

Least square means values of DON analyzed with GC and Liquid chromatography—mass spectrometry (LC-MS), D3G determined with LC-MS, and percent damage kernels are given in Table 2. These variables showed significant differences between state and region mean values, indicating that HRS wheat samples collected from different state or regions might have different levels of DON and D3G. Samples collected from ND presented higher values of DON (both GC and LC-MS methods), D3G, and percent damaged kernels than other states in both years. Samples from MT presented the opposite trend. The regional data showed more specifically that samples collected from ND contained higher levels of DON, D3G and percent damaged kernels than the other states. DON, D3G and percent damaged kernels presented higher values for samples collected from ND regions A, B, C, E and F in 2011. For ND samples collected in 2012, DON, D3G and Kernel damaged showed higher values only for samples from regions A and B. Among MN regions, samples collected in 2011 from region B showed high DON and percent damaged kernels. Samples from SD region B in 2011 also showed high levels of DON, D3G and percent kernel damage. For the 2012 samples, those collected from MN region A and MT region B showed high D3G values. Specifically, samples from MT region B showed high D3G content despite low DON content in 2012. The rest of the regions presented similar content of DON, D3G and percent damaged kernels and the majority did not show significant differences. The 2011 survey samples had high DON content, independent of the method used. Due to the high levels of DON during this year, the percent damaged kernels were also higher compared to 2012 survey samples. 

**Table 2:** Least square mean values for deoxynivalenol (DON), deoxynivalenol-3-glucoside (D3G) and damage kernel in 2011 and 2012 survey.

Growing Area	2011				2012			
State	GC-DON	LC-DON	D3G	Damage (%)	GC-DON	LC-DON	D3G	Damage (%)
MN	1.35 **	1.74 **	0.04	0.35 **	0.89	0.78	0.128 **	0.05
MT	0.03	0.03	0.00	0.03	0.18	0.18	0.090	0.00
ND	2.80***	3.15 ***	0.24 ***	0.63 ***	1.93 ***	1.71 ***	0.142 ***	0.06 ***
SD	1.35 **	1.72 *	0.12	0.36 *	0.37	0.33	0.085	0.03
**Region**								
MN-A	1.07	1.21	0.01	0.23	0.82	0.72	0.124 *	0.08 *
MN-B	1.63 *	2.27 **	0.06	0.47 *	0.96	0.84	0.133	0.02
MT-A	0.00	0.00	0.00	0.01	0.09	0.12	0.067	0.01
MT-B	0.00	0.00	0.00	0.05	0.04	0.05	0.176 **	0.00
MT-C	0.13	0.13	0.00	0.03	0.11	0.20	0.105	0.00
MT-D	0.00	0.00	0.00	0.08	0.00	0.08	0.015	0.00
MT-E	0.04	0.00	0.00	0.00	0.65	0.47	0.088	0.00
ND-A	3.89 ***	4.26 ***	0.31 ***	0.75 ***	6.91 ***	5.97 ***	0.295 ***	0.15 ***
ND-B	3.77 ***	4.32 ***	0.30 ***	0.69 ***	2.48 ***	2.22 ***	0.174 ***	0.12 ***
ND-C	2.91 ***	3.36 ***	0.30 ***	0.61 ***	0.66	0.60	0.060	0.06
ND-D	1.08	1.10	0.02	0.61 ***	0.34	0.35	0.094	0.01
ND-E	2.19 **	2.54 **	0.09	0.66 ***	0.71	0.72	0.096	0.02
ND-F	3.00 **	3.34 ***	0.39 ***	0.49 **	0.47	0.38	0.133	0.01
SD-A	0.21	0.15	0.00	0.13	0.49	0.42	0.094	0.00
SD-B	2.41 ***	3.20 ***	0.29 **	0.57 **	0.22	0.23	0.097	0.00
SD-C	1.42	1.82	0.06	0.38	0.41	0.34	0.066	0.09

Notes: *, **, and *** means significant differences (H0: least square mean = 0) at *p* ˂ 0.05, 0.01, and 0.001, respectively. Values of DON and D3G are in mg/kg; gas chromatographic (GC).

D3G content in 2011 was not different among states except for ND, but in 2012, MN also showed differences in D3G content. Also, the differences in DON content and D3G among regions and states can be attributed to the environmental conditions presented in each year of survey. The analysis of variance (ANOVA) indicates that state and region had significant effects on variation in DON and D3G content and percent damaged kernels in 2011 survey samples (Table 3). The ANOVA for 2012 survey samples showed slightly different results (Table 3). During 2012 the percent damaged kernels was not affected by the state, region or their interaction. The ANOVA on DON and D3G for both years indicates that the wheat growing environment greatly affects the variations in DON and D3G content. Schmidt-Heydt *et al.* reported that the key genes in the biosynthetic pathway of mycotoxin production could be influenced by the environmental factors and water activity [4]. Therefore, the gene expression resulted in different effects, depending on their interaction with the abiotic conditions, on the DON production levels. On the other hand, D3G is produced by the plant as a detoxification process in response to the DON production [11]. So, it could be possible that the D3G content among states may depends of the infection level of DON in the wheat.

**Table 3:** Mean square values of state (ST), region (Rga) and county (CTY) on DON, D3G and damaged kernel in 2011 and 2012 survey.

Year	Source	Degrees of freedom	Mean square			
			GC-DON	LC-DON	D3G	Damage
2011	ST	3	221.3 ***	282.8 ***	2.15 ***	10.19 ***
	Rga (ST)	12	28.0 **	40.4 ***	0.43 *	0.45
	CTY (Rga × ST)	100	8.9	13.2	0.19	0.44
	Residual	320	9.7	14.0	0.24	0.70
2012	ST	3	151.2 ***	111.2 ***	0.15 ***	0.22 **
	Rga (ST)	12	104.3 ***	72.8 ***	0.12	0.12 ***
	CTY (Rga × ST)	100	14.5	11.3	0.07	0.04
	Residual	320	12.0	10.9	0.07	0.04

Notes: *, **, and *** means F values are significant at *p* ˂ 0.05, 0.01, and 0.001, respectively.

### 2.3. Correlation among DON, D3G with Percent Damaged Kernels

**Figure 2:**
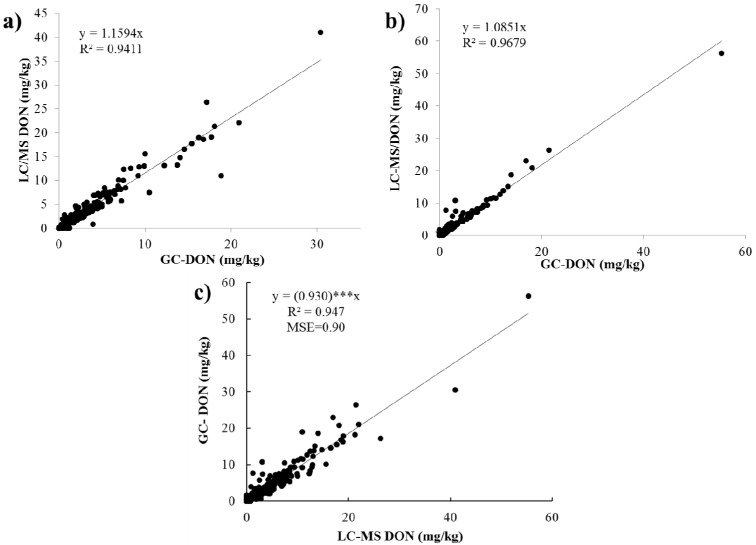
Correlation GC-DON and LC-MS DON content values. (**a**) 2011 survey samples; (**b**) 2012 survey samples and (**c**) 2011 and 2012 survey samples combined. *** Significantly different from 1 at *p* < 0.001.

**Figure 3:**
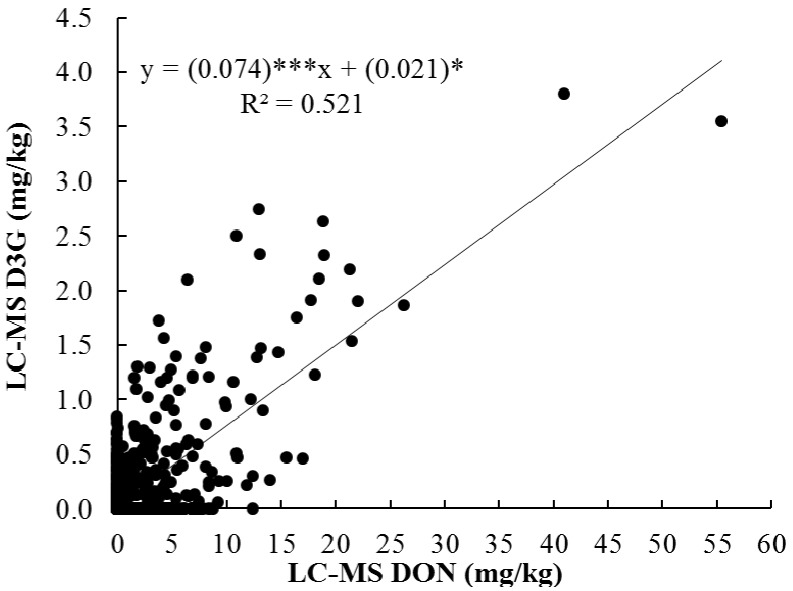
Correlation between DON and D3G levels in survey samples between 2011 and 2012; where ***, and * indicate that regression coefficients are significant at *p* < 0.001 and *p* < 0.05, respectively.

The correlation between GC and LC-MS methods used to analyze DON during 2011 and 2012 is shown in Figure 2a,b. DON was measured by both GC and LC-QTOF-MS to evaluate the correlation between methods and determine the feasibility if the LC-QTOF-MS methodology for simultaneous measurement of DON and D3G. The high coefficient of determination (R^2^) indicates strong, positive and significant correlation between both methods in both years (Figure 2). When the GC and LC-MS methods for both survey 2011 and 2012 were compared (Figure 2c), an R^2^ of 0.947 and mean square error (MSE) of 0.90 were found. To identify relationships between mycotoxin contents and percent damaged kernels, linear and rank correlation coefficients were estimated and given in Table 4. The DON values determined GC and LC methods also showed very high and positive correlation for 2011 and 2012 data, individually (Table 4). These results mean that the LC method is as precise as or better in evaluating DON concentration of HRS wheat lines when compared to the GC method. The linear and rank correlations were significant (*p* < 0.01) and positive between DON concentration data determined for 2011 and 2012 samples. This result indicates that year by region interaction might not be strong, and the region that had higher DON concentration in 2011 samples also had higher DON concentration than other regions in 2012. Specifically, ND regions A and B showed higher DON concentration than other regions for both 2011 and 2012 samples. Further research may be needed to verify this since current results are based on data collected from only 2 years. The correlation between DON and D3G (Figure 3) in survey samples between 2011 and 2012 was significant with a moderate *R^2^* = 0.521. This means that the D3G production is related positively to the DON content and increasing DON levels also increase the D3G level in wheat. This is in agreement with the results from other researchers [10,13]. However, the moderate R^2^ value indicates that DON concentration was partially responsible for D3G variation in this sample set. This is due to the low correlation between DON and D3G for 2012 samples (Table 4). To be more specific, 2012 samples from MN region A and MT region B had high D3G concentrations despite low DON concentrations (Table 2). These results indicate that, for precise evaluation of mycotoxin in HRS wheat samples, the LC-MS method is better to use for determination of DON and D3G content rather than the GC method which can only determine DON.

**Table 4:** Pearson linear and Spearman rank correlation coefficients between DON, D3G and damage kernel for regions.

Year	Variables	GC-DON	LC-DON	D3G	Damage
		Linear correlation			
2011	GC-DON	-	0.99 ***	0.91 ***	0.91 ***
	LC-DON	0.99 ***	-	0.91 ***	0.91 ***
	D3G	0.94 ***	0.92 ***	-	0.74 **
	Damage	0.89 ***	0.89 ***	0.87 ***	-
		Rank correlation			
		Linear correlation			
2012	GC-DON	-	1.00 ***	0.83 ***	0.79 ***
	LC-DON	0.99 ***	-	0.83 ***	0.79 ***
	D3G	0.41^NS^	0.37^NS^	-	0.60 *
	Damage	0.72 **	0.69 **	0.28^NS^	-
		Rank correlation			

Notes: *, **, and *** means correlation coefficient is significant at *p* ˂ 0.05, 0.01, and 0.001, respectively. NS: Not significant (*p* ≥ 0.05).

**Figure 4:**
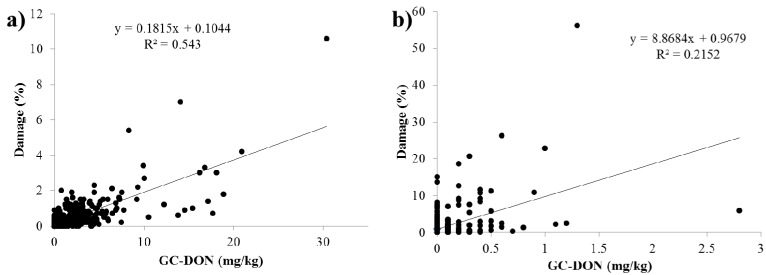
Correlation between GC-DON and damage levels in survey samples from (**a**) 2011 and (**b**) 2012.

The correlation among DON and D3G with damage kernel during 2011 and 2012 is given in Table 4 and Figure 4. The percent damaged kernels had very highly significant correlation with GC-DON and LC-DON (*p* < 0.001) in 2011; damage also had a very highly significant correlation at *p* < 0.01 with DON (GC and LC) in 2012. The positive correlations indicate that samples which were rated to have higher percent damaged kernels had higher levels of DON in the sample. This was also shown in Figure 4a,b, where the scatter plot between GC-DON and damage was depicted. D3G also had a significant (*p* < 0.05) correlation with percent damaged kernels in 2011 and 2012. This indicates that as the *Fusarium* infection progresses more kernel damage occurs. While DON levels rise which leads to increased production of D3G as the plants detoxification mechanism. 

## 3. Experimental Section

### 3.1. Standards and Chemicals

DON (100.2 μg/mL) and D3G (50.2 μg/mL) both in acetonitrile were purchased from Biopure (Tulln, Austria). For the gas chromatography–electron capture detection (GC–ECD) method, 5 mg/L DON working standard solution was used to make a standard curve prepared in a clean wheat extract. In the LC-MS method, stock solutions were dissolved in acetonitrile and stored in the refrigerator. Composite working standard solutions were prepared by dilution of the stock solutions in a DON-free wheat matrix to prepare matrix-matched calibration standards in concentration of 0 to 20 mg/L for DON and 0 to 10 mg/L for D3G. Acetonitrile was purchased from J. Baker. TMSI (1-(trimethylsilyl)imidazole), TMCS (Chlorotrimethylsilane) and 2,2,4-trimethylpentane (ACS reagent) were obtained from Sigma Aldrich. 

### 3.2. Samples

The hard red spring (HRS) wheat between 2011 and 2012 Crop Survey samples were used as raw material. A total of 441 and 436 samples were selected as wheat grader samples from the 2011 and 2012 HRS wheat crop surveys, respectively, and used in this study. The samples were collected based on production data obtained from the National Agricultural Statistics Service for the 16 regions in the 4 state HRS wheat growing region (Figure 1). The Montana (MT), North Dakota (ND), South Dakota (SD) and Minnesota (MN) state office of the National Agricultural Statistics Service obtained wheat samples during harvest directly from growers either in the fields or farm bins and local elevators. Samples from the 2011 Crop Survey represented a high FHB infection and incidence of DON, while 2012 Crop Survey samples represented wheat with low FHB infection. 

### 3.3. Wheat Kernel Quality

The kernel quality based on non-grading factors consisted of the determination of the protein content (expressed in 12% moisture basis, Method 39.10.01), falling number expressed in seconds (Method 56.81.03), both approved methods of the AACC [21] and thousand kernel weight determined on a 10 g sample of cleaned wheat (free of foreign material and broken kernels) counted by electronic seed counter.

The wheat grade and class of the samples was determined by a licensed grain inspector for the Official United States Standards for Grain. North Dakota Grain Inspection Service, Fargo, ND, provided grades for composite wheat samples. The final grade of the samples was based on dockage (elimination of all material other than wheat), shrunken and broken kernels and percent damaged kernels, test weight measured as pounds per bushel (l b/bu) (Method 55-10, AACC) and percent vitreous kernels (percentage of kernels having vitreous endosperm), as well as the summation of these defects referred to as total defects using an official procedure of USDA (United Stated Department of Agriculture). 

### 3.4. Sample Preparation for GC–ECD and LC-MS

#### 3.4.1. Free DON Analysis with GC–ECD

**Figure 5:**
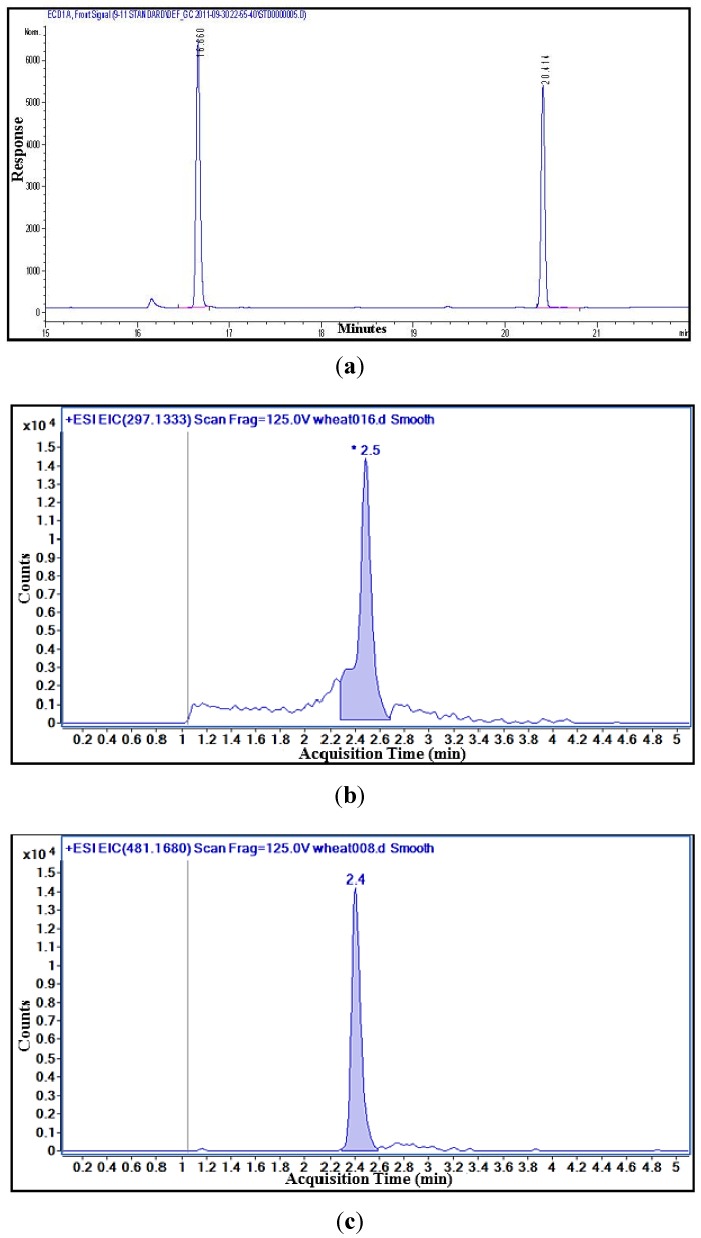
Representative chromatograms of a wheat sample containing approximately 2.0 mg/kg DON and 1.0 mg/kg D3G. (**a**) gas chromatography–electron capture detection (GC-ECD) chromatogram of DON * and Mirex ** (internal standard); (**b**) LC-MS extracted ion (m/z of (M + H) + ion 297.1333) chromatogram of DON; and (**c**) LC-MS extracted ion (m/z of the (M + Na) + ion 481.1680) chromatogram of D3G.

Free DON was determined using the methodology described by Tacke *et al.* [22] with some modifications [2]. One g of sample was dispersed in 8 mL of acetonitrile:water mixture (84:16, v/v) and shake for 1 h at 180 rpm. The extract (4 mL) was passed through cleanup column (Extract clean^TM^ C18-Al, GRACE, Illinois, USA) and 2 mL of the filtrate were evaporated until dryness with nitrogen at 55 °C. Then, 100 μL of TMSI-TMCS (100:1, v/v) was added to the solution to residue. The samples were vortexed for 10 s and allowed to react for 5 min at room temperature. The internal standard solution (1 mL, 0.5 mg/L Mirex) was added and swirled. Immediately after which, 1 mL of water was added and the tube was shaken for 5 min. The samples were left at room temperature until two layers were observed. The top layer was transferred to vials for analysis by gas chromatography–electron capture detection (GC–ECD) with a series of standards. The GC–ECD equipment was Agilent 6890 GC with dual injector, cool on-column inlet, HP-5 column (30 m, 0.25 mm and 0.25 μm) and ECD detectors (Agilent Technologies, Wilmington, USA). Helium and argon-methane were used as carrier and makeup gas, respectively. The DON recoveries as determined by Tacke *et al.* [22], were 100%, 94% and 94% for 0.5, 4.0 and 20.0 mg/kg DON, respectively. The limit of detection (LOD) was determined to be 0.05 mg/kg and the limit of quantitation (LOQ) was determined to be 0.2 mg/kg [22]. Figure 5a shows a reference chromatogram of 2.0 mg/kg DON determined by GC–ECD.

#### 3.4.2. Free DON and D3G Analysis with LC-MS

The sample preparation was carried out according to Tacke *et al.* [22] with a few modifications. The sample (2.5 g) was extracted with 20 mL of acetonitrile/water mixture (84:16; v/v) for 1 h on an orbital shaker at 180 rpm. The samples were left 20–30 min to settle. One mL of the crude extract was filtered with 0.2 μm nylon syringe filter into a glass vial. The sample was analyzed with a liquid chromatographic coupled with a quadrupole time of flight system (LC-QTOF). A series of DON and D3G standards were also prepared and analyzed with every set of samples run on the LC-QTOF. Matrix matched calibration standards were prepared at several concentrations for DON and D3G to take into consideration any matrix effects from compounds extracted from the wheat. The concentrations of DON were 20.0, 10.0, 6.0, 4.0, 2.0, 1.0, 0.7, 0.5, 0.2 and 0.1 mg/kg and the D3G concentrations were 5.0, 3.0, 2.0, 1.0, 0.75, 0.5, 0.3 and 0.2 mg/kg. Figure 5b,c shows reference chromatograms of DON (2.0 mg/kg) and D3G (1.0 mg/kg) determined by LC-MS.

#### 3.4.3. LC-MS Instrumentation and Methodology

The analysis of mycotoxins by liquid chromatography mass spectrometry (LC-MS) was performed according to the methods of Vendl *et al*. [23] and Simsek *et al.* [2] with some modifications. A 1290 UPLC System (Agilent Technologies, Wilmington, DE, USA) was used for separation of analytes. The DON and D3G separation was carried out with an Eclipse Plus C18 column (Zorbax Rapid Resolution High Definition (RRHD), 2.1 × 100 mm, 1.8-Micron, Agilent Technologies, Wilmington, DE, USA). The column temperature was set to 40 °C. The solvent system consisted of 0.1% formic/water (solvent A) and 0.1% formic/acetonitrile (solvent B). Formic acid was used instead of acetic acid to improve formation of the M+H ion rather than formation of M+Na ion found frequently when using acetic acid. The purge was done with 100% A with a purge flow rate of 4 mL/min during 15 s and the isocratic pump flow was 0.6 mL/min with 100% A. The solvent gradient was modified slightly to reduce run time and improve chromatographic separation of DON and D3G. The gradient program started with 97% A and 3% B with a binary pump flow rate of 0.4 mL/min and was kept until 0.75 min. Afterwards, the proportion of B was increased linearly to 100% within 4 min, followed by a hold time of 6 min at 100% B and 10 min re-equilibration at 97% A, followed by isocratic washout step for 2 min with 97% A. Five μL was the volume injection used. 

The analyte detection was determined with a mass spectrometer quadrupole time of flight (Agilent 6540 time-of-flight LC-MS, Agilent Technologies, Wilmington, DE, USA). The ESI interface was used in positive-ionization mode at 300 °C with the following settings: 7 L/min gas flow, 30 psig nebulizer gas, 225 sheath gas temperature and 12 of sheath gas flow. Acquisition mode MS1 parameters were minimal range (m/z) 100, maximum range (m/z) 1700 and scan range of 2 spectra/s. The data analysis was performed using a MassHunter Qualitative Analysis B.05.00 program (Agilent Technologies, Wilmington, USA). Integration and calculation of DON and D3G were prepared by extracting the ion chromatograms for DON and D3G from the total ion chromatogram (TIC) using a mass window of 10 ppm. The DON extracted ion chromatogram used the m/z of the (M+H)^+^ ion (297.1333) and the D3G EIC used the *m/z* of the (M+Na)^+^ ion (481.1680). Since the detection method was TOF-MS without fragmentation the accurate mass and retention time of standards were used for identification of compounds (DON and D3G) as determined by Vendl *et al.* [23]. The DON recovery was calculated to be 107% whole the D3G recovery was 51% [23].

### 3.5. Statistics Analysis

Analysis of variance (ANOVA) was performed for individual years using the “MIXED” procedure in SAS (V 9.2, SAS Institute Inc., Cary, NC, USA). The model for ANOVA was a nested fixed model in which region was nested in state and city was nested in region. Least square mean values were estimated using the “LSMEAN” option. Correlation and regression was performed using “CORR” and “GLM” procedures in SAS, respectively.

## 4. Conclusions

The results indicated that DON levels varied with the survey crop year and they have a relationship with the kernel quality and D3G detected in wheat. Also, it was found that the growing state cause a larger effect on DON and D3G, but not on percent damaged kernels. The D3G levels were significantly correlated with the percent damaged kernels, but at lower levels than the DON content. DON infection in wheat caused more effect on the kernel quality between years analyzed. Otherwise, the ANOVA and correlation coefficient indicate that both GC and LC-MS can be used to determine DON in HRS. However, due to the ease of the method (sample can be extracted and analyzed without derivatization) and simultaneous determination of the D3G, LC-MS is more advantageous.
